# Nature and Lability of Northern Adriatic Macroaggregates

**DOI:** 10.3390/md8092480

**Published:** 2010-09-06

**Authors:** Jadran Faganeli, Bojana Mohar, Romina Kofol, Vesna Pavlica, Tjaša Marinšek, Ajda Rozman, Nives Kovač, Angela Šurca Vuk

**Affiliations:** 1 Marine Biological Station, National Institute of Biology, 6330 Piran, Slovenia; E-Mails: moharbojana@yahoo.co.uk (B.M.); rkofol@gmail.com (R.K.); vesna.pavlica@gmail.com (V.P.); tjasa.marinsek@gmail.com (T.M.); ajdaroz@yahoo.co.uk (A.R.); kovac@mbss.org (N.K.); 2 National Institute of Chemistry, Hajdrihova 19, 1000 Ljubljana, Slovenia; E-Mail: angela.surca.vuk@ki.si

**Keywords:** marine macroaggregates, lability, northern Adriatic

## Abstract

The key organic constituents of marine macroaggregates (macrogels) of prevalently phytoplankton origin, periodically occurring in the northern Adriatic Sea, are proteins, lipids and especially polysaccharides. In this article, the reactivity of various macroaggregate fractions in relation to their composition in order to decode the potentially »bioavailable« fractions is summarized and discussed. The enzymatic hydrolysis of the macroaggregate matrix, using α-amylase, β-glucosidase, protease, proteinase and lipase, revealed the simultaneous degradation of polysaccharides and proteins, while lipids seem largely preserved. In the fresh surface macroaggregate samples, a pronounced degradation of the α-glycosidic bond compared to β-linkages. Degradation of the colloidal fraction proceeded faster in the higher molecular weight (MW) fractions. *N*-containing polysaccharides can be important constituents of the higher MW fraction while the lower MW constituents can mostly be composed of poly- and oligosaccharides. Since the polysaccharide component in the higher MW fraction is more degradable compared to *N*-containing polysaccharides, the higher MW fraction represents a possible path of organic nitrogen preservation. Enzymatic hydrolysis, using α-amylase and β-glucosidase, revealed the presence of α- and β-glycosidic linkages in all fractions with similar decomposition kinetics. Our results indicate that different fractions of macroaggregates are subjected to compositional selective reactivity with important implications for macroaggregate persistence in the seawater column and deposition.

## 1. Introduction

The northern Adriatic “mucilage phenomena”, or mucous macroaggregates, usually develop in late spring or early summer. Although their formation is not yet completely understood, these events seems to be significantly linked to preceding changes of the seawater inorganic N/P ratio [1,2] which can influence the rate of phytoplankton growth and phytoplankton metabolism inducing the production of mucilaginous material [3–5]. The phytoplankton origin is also indicated by mucilage composition, mostly composed of heteropolysaccharides produced by phytoplankton exudation [6] and cell lyisis [7]. Microscopic observations [7–11] indicate diatoms as their crucial producers although other phytoplankton species and bacteria are also present in macroaggregates. Concentrations of nutrients present during the marked retention of freshened waters and water column stratification in the northern Adriatic [12] seem to be less critical for mucous formation. Specific oceanographic conditions, including the formation of a gyre, higher seawater residence time, and development of a marked pycnocline during stable summer conditions with low turbulent shear [13], significantly contribute to the subsequent concentration and agglomeration of macromolecular organic matter, phytoplankton cells and other organics and minerals. The aggregation process can be explained by polymer gel theory [14] through the formation of nanogels, later microgels which continue to aggregate into macrogels and POM [15,16]. Macroaggregates represent basically the transition from colloidal organic matter (COM, macromolecules) into macrogels and particulate organic matter (POM). Their gel-like nature is manifested by a high content of water, sometimes exceeding 95%, and organic matter which represents about 35–57% of dry mass. The organic component is mostly composed of carbohydrates (12–34%), proteins (1–12%) and lipids (0.1–8%)[17] and the inorganic fraction is predominantly composed of quartz, calcite and clays. The latter generally results from the scavenging and inclusion of autochthonous and allochthonous particles [11] from the ambient seawater. The association of inorganic and organic particles [18] and that of colloids with cations [15] probably contributes to the stabilization and persistence of those macrogels in the aquatic environment.

Mucous macroaggregates can be observed in various forms, in the surface layer, in the water column and on the seabed. Their different color, size and shape depends on their biological and chemical composition, maturation stage, *i.e.*, age, basic water-column characteristics and environmental conditions, as well as local hydrodynamics [9,10,19–21]. Macroaggregates are continuously subjected to microbial and various chemical and photochemical transformations [17]. However, the bacteria are believed to be a principal decomposer of the macroaggregate biopolymers [21,22] leading to low molecular weight products finally released to marine dissolved matter. Complete disappearance of macroaggregates, mostly in the late summer, usually coincides with rain storm events, changes in the water column stratification and circulation pattern [23].

The principal aim of this article was to present our recent investigations on the reactivity of various macroaggregate fractions, namely matrix and interstitial water colloids, in relation to their composition as well as the linkage of macroaggregate structural units using laboratory based enzymatic and natural degradation approaches.

## 2. Results and Discussion

### 2.1. Macroaggregate composition: matrix

**Figure 1:**
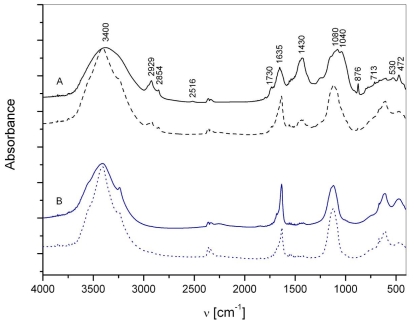
FT-IR spectra of (**A**) macroaggregate matrix, surface sample (black bold line) and water column sample (black dotted line); and (**B**) macroaggregate interstitial water colloids, surface sample (blue bold line) and water column sample (blue dotted line).

The matrix can be viewed as the water-insoluble fraction of macroaggregates. Major spectrum bands (Figure 1A) could be assigned: 3000–3600 cm^−1^ (O-H, N-H stretching band region), 3000–2800 cm^−1^ (aliphatic groups), 1730 cm^−1^ (C=O stretching of COOH, ketones or aldehides), 1650–1640 cm^−1^ (C=O stretching of amide I; C=N stretch; stretching of COO^−^; C=C signals of aromatic ring and/or olefins C=C, water deformational modes), 1430 cm^−1^ (aliphatic C-H deformation of CH_2_ and CH_3_ groups; symmetric stretching of COO^−^), 1150–1000 cm^−1^ (carbohydrates, Si-O stretch). Spectra of surface matrix display also main calcite peaks situated at 2516, 1420–1450, 876 and 713 cm^−1^ and that of silicates (527–535 and 472 cm^−1^). The elemental composition of surface and water column samples revealed higher values for surface macroaggregates, averaging 16.4% of C_org._, 0.7% of N_tot._ and 254 ppm of P_tot._, with a mean C:N:P ratio (molar) of 772:26:1. Accordingly, higher contents of total carbohydrates and proteins, averaging 13.5% and 4.7%, respectively, were determined in surface samples compared to deeper seawater layer samples. This statement is supported also by FTIR analyses (Figure 1) showing the presence of carbohydrates, evidenced by bands at ~1150–900 cm^−1^ and proteins, evidenced by bands at 1654–1635 cm^−1^, in both samples. The lipid contents, clearly indicated by bands at 2950–2850 cm^−1^ in FTIR spectra (Figure 1A), are lower, averaging 2.2%.

### 2.2. Macroaggregate composition: interstitial water colloids

**Figure 2:**
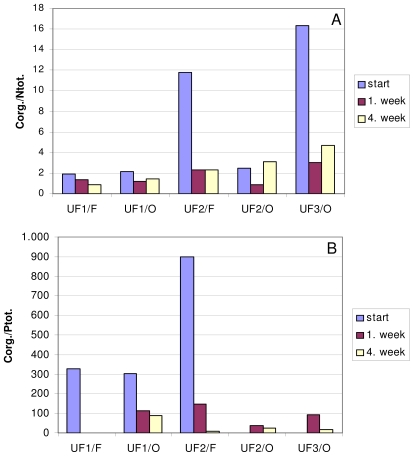
C_org._/N_tot._ (**A**) and C_org._/P_tot._ (**B**) molar ratios ultrafiltrate retentates (UF/0) and permeates (UF/F) using a nominal molecular weight cutoff of 30–10 (UF1), 10–5 (UF2) and <5 kDa (UF3) at the start, after 1 week and after 4 weeks of the degradation experiment.

FTIR spectra of interstitial water colloids (the “water-soluble” fraction) show the presence of both basic constituents, *i.e.*, carbohydrates and proteins (Figure 1B). In FT-IR spectra of all colloidal fractions (not shown), the presence of a band at 2240 cm^−1^ probably indicates the cyanogenic glycosides [24] represents the greatest part of organic nitrilated compounds [25]. Their existence was previously confirmed also by monosaccharide analyses in bulk macroaggregates [26]. Their source is most probably linked to amino acids [25]. The bands assigned to lipids, *i.e.*, aliphatic components, at 2800–3000 cm^−1^ are less evident. The important contribution of carbohydrates in all colloid fractions was supported by higher UV absorption at λ = 250 nm, more typical of polysaccharides, compared to λ = 205 nm, being more typical of proteins [27] and humics [28]. The highest carbohydrate content and the lower C/N and C/P ratios (Figure 2) were found in the higher molecular weight (MW) colloidal fraction indicating the important presence of N and P containing [29] carbohydrates as well as their important role in mucilage composition. The δ^15^N data exhibiting low values, especially in the >30 kDa fraction [30] and previous reports on non-protein nitrogen compounds [31], also sustain the presence of non-protein N compounds, *i.e.*, amino sugars, chlorophyll and nucleic acids, in the colloidal fraction. In contrast, the lower MW fractions seem mostly composed of oligo- and polysaccharides.

## 3. Macroaggregate Lability

### 3.1. Matrix

**Figure 3:**
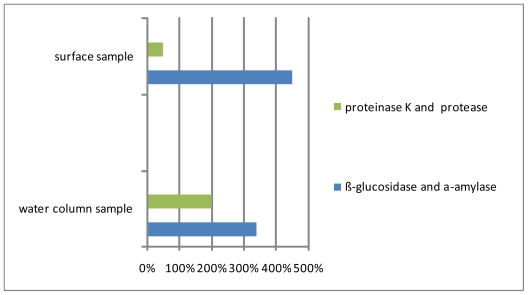
Concentration changes of carbohydrates and proteins during various enzyme hydrolyses (6 hours at 26 °C).

According to macroaggregate enzymatic hydrolysis with α-amylase and β-glucosidase, the α (1–4) reserve polysaccharides, abundantly present in dinofllagelates in surface bulk macroaggregate samples taken in July 2004, are the most susceptible for enzymatic hydrolysis. In contrast, in the water column macroaggregates, containing prevalently diatoms, the impact of α-amylase is lower (Figure 3).

**Figures 4:**
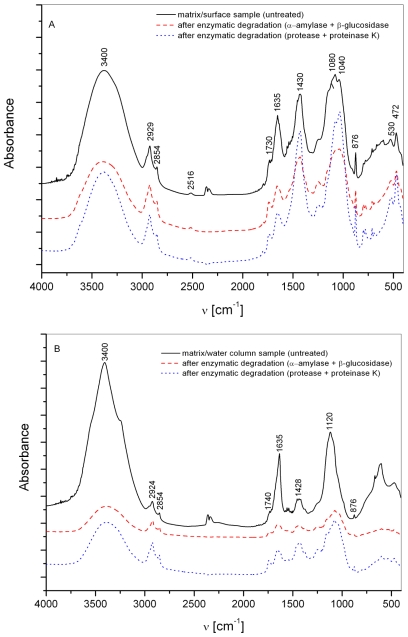
FT-IR spectra of the (**A**) surface and (**B**) water column macroaggregate matrices, and after (i) α-amylase + β-glucosidase, (ii) protease + proteinase K hydrolysis: carbohydrate bands (region ~1150–900 cm^−1^), protein bands (region 1654–1635 cm^−1^), lipid bands (region 2950–2850 cm^−1^) and inorganic (mineral) components (region <1000 cm^−1^).

The presence of α- and β-glycosidic linkages in macroaggregates was previously confirmed by ^1^H-NMR spectroscopy [32] and a shift from α- to the more refractory β-glycosidic linkage was described in aged macroaggregates dominated by diatoms containing storage β-glucans [19,22]. These findings are in accordance with the observations of Herndl *et al.* [33] and Zaccone *et al.* [34] who found that the activities of α- and β-glucosidase in macroaggregates were opposite due to microbial response to the composition of substrates, *i.e.*, macroaggregate components, where β-glycosidic linkage seems more refractory. The compositional difference between “fresher” surface and aged water column macroaggregate samples is also indicated by results of enzymatic hydrolysis (Figure 4). The addition of α-amylase and β-glucosidase produces high carbohydrate release in both samples (Figure 4A and 4B). Protease and proteinase K, the most active ectoenzymes found in particles [35] and macroaggregates [22], produce, in addition to proteins, higher carbohydrate release in the water column sample which is clearly evident from FTIR spectra (Figure 4B) showing a greater decrease in the carbohydrate band at ~1150–1000 cm^−1^.

**Figures 5:**
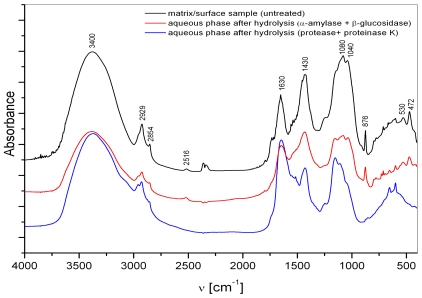
FT-IR spectra of the surface macroaggregate matrix and aqueous phase of experimental slurries after α-amylase + β-glucosidase and protease + proteinase K hydrolysis.

FTIR spectra of the water phase from macroaggregate matrix slurries, obtained after hydrolysis with α-amylase + β-glucosidase, indicate the presence of minerals (Figure 5) such as calcite (signals at 2516, 1430, 876 cm^−1^) and silicates (530 and 472 cm^−1^). These results confirm the importance of associations between carbohydrates and minerals (inorganic species) for mucilage events. The release of minerals was not observed in the case of the same treatment of more mature water column sample, probably due to more intense interactions between organic and inorganic components; the latter acting as a stabilizing agent.

In aged macroaggregates, the carbohydrates can be linked to proteins, probably in the form of glycoproteins, and thus the organic N can be preserved in these probably more crosslinked samples [36]. The rapid hydrolysis of organophosphorus in the macroaggregate matrix by phosphatase, a nearly 300% increase of phosphate concentrations in the first ten minutes, suggests faster cycling of P with respect to N. Phosphatase can also be involved in the hydrolysis and release of organic constituents [37].

**Figure 6:**
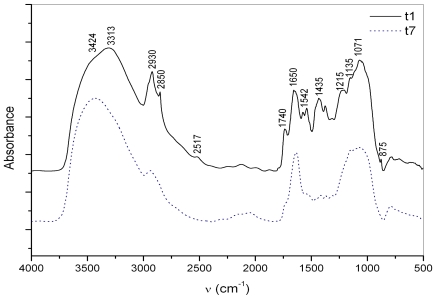
FT-IR spectra of the surface macroaggregate matrix (sampled at the beginning of the degradation experiment; t_1_—black line), and after lipase (after 3 weeks at 26 °C, t_1_—blue line) hydrolysis: carbohydrate bands (region ~1150–900 cm^−1^), protein bands (region 1654–1635 cm^−1^), lipid bands (region 2950–2850 cm^−1^) and inorganic (mineral) components (region <1000 cm^−1^).

The hydrolysis of surface (fresher) macroaggregates with lipase showed primarily the degradation of lipids and polysaccharides, suggesting an association of lipids with polysaccharides possibly in the form of glycolipids. Using lipase (Figure 6) the macroagregate degradation is slower, ranging from days to weeks, compared to proteinase K and protease, ranging from hours to days. In the three week long degradation experiment, the relative intensities of absorptions indicating the lipidic component (2800–3000 cm^−1^, 1730–1740 cm^−1^, 1430–1450 cm^−1^), revealed a large decrease in lipids, only at the end of the experiment due to their lower degradability.

The previous studies [18,36] of temporal compositional changes of the northern Adriatic bulk macroaggregates in the summer stratified seawater column, using ^1^H- and ^13^C-NMR and FTIR spectroscopy, also revealed the preferential degradation of carbohydrates in comparison to the aliphatic components—lipids. Similar results were obtained in the spectroscopic ^1^H-NMR study of the bulk macroaggregates, showing much faster degradation of carbohydrates compared to lipids during the mucilage event [31]. All these findings seem to contradict the reported observations of the high hydrolytic activity of lipases, one of the most active ectoenzymes in aquatic systems [38] and in macroaggregates [39].

Regardless of bacterial enzymatic activity, the macroaggregate hydrogel structure can slow microbial degradation [40]. The gel microhabitat can represent an important site of organic matter degradation (microbial functioning) but, on the other hand, the gel polymers can be preserved due to their sterical hindrance [40] and the presence of organic-inorganic associations.

### 3.2. Interstitial water colloids

The enzymatic hydrolysis of all colloidal fractions using α-amylase and β-glucosidase indicates the presence of α- and β-gycosidic linkages in nearly equimolar proportions. Similar kinetics of hydrolysis appears in the >30 kDa and 10–30 kDa fractions. In the 5–10 kDa fraction, the increase of carbohydrate content after seven days is probably due to the prevalently polysaccharidic nature of this fraction making it more suitable for enzymatic hydrolisis. Contents of C_org._, N_tot._and P_tot._ and the results of HPSEC analyses [41] from the »natural« degradation of macroaggregate colloidal fractions, indicate faster degradation in the >30 kDa and 30–10 kDa fractions compared to the 10–5 kDa fraction. This could be due to the higher contents of *N*-containing polysaccharides in the higher MW fractions. The microbial degradation of the carbohydrate component of glycoproteins is reported to proceed up to 3-fold faster compared to the proteinaceous components [42,43] explaining the preservation of organic nitrogen and the decreasing C/N ratio in aged degraded mature macroaggregates.

## 4. Experimental Section

### 4.1. Samples

Macroaggregate samples were collected on 1 July, 2004, in the southern part of the Gulf of Trieste at a fixed sampling site (45° 31.46′ N, 13° 33.72′ E) at the sea surface and at a depth of 10 m above the pycnocline. The sea water temperature was 25 °C. Both macroaggregate samples were in macrogel form; hence it was possible to collect them by hand by SCUBA divers, using polyethylene bottles with a minimal amount of surrounding water. The macroaggregate interstitial water was isolated by filtration through a 50 μm mesh size plankton net and centrifuged at 4000 g. The supernatant was successively filtered through preignited 0.7 μm pore size Whatman GF/F filters. The sediment (water-insoluble fraction) experiments and filtered supernatant (water-soluble fraction) were freeze-dried and used for subsequent degradation and fractionation, and degradation experiments, respectively.

### 4.2. Degradation experiments and separations

#### 4.2.1. Matrix

The dry macroaggregate matrix was diluted with distilled water (aqueous phase) to obtain aqueous slurries of water-insoluble macroaggregate matrix. It was further enzymatically hydrolyzed for 0, 5, 10, 30, 60, 120, 240 and 360 min at 26 °C using 0.5 mL of α-amylase, β-glucosidase, protease or 1 mL proteinase K (all Sigma). The concentrations of all used aqueous enzyme stock solutions were 1 mg/mL. Enzymatic hydrolisis with 0.2 mL of lipase (Sigma) at 26 °C lasted for 0, 20, 60 min, and 1, 7, 14 and 21 days. The concentration of the used lipase stock solution was 6 mg/mL. The hydrolyzates were successively centrifuged at 3000 g, and the supernatant and sediment used for determination of total carbohydrates, proteins and lipids, and FTIR analyses. The blanks, consisting of samples without the addition of enzymes, represented substrates in a »natural« degradation experiment. All experiments were performed in triplicate.

#### 4.2.2. Interstitial water colloids

In the natural degradation study, the filtrate was incubated for four weeks at 26 °C in the dark and subsamples were taken at the start (t = 0), after one (t = 1), two (t = 2), three (t = 3) and four weeks (t = 4). The subsamples were subsequently filtered through 0.22 μm pore size Nucleopore filters and separated by ultrafiltration. Ultrafiltration in a “cascade fashion” through membranes with nominal molecular weight cutoff (MWCO) values of 30, 10 and 5 kDa was performed using a Vivascience VivaFlow 200 unit (Sartorius) with a MasterFlex L/S membrane pump (Cole-Palmer) at a flow rate of 300 mL min^−1^ at 2.5 bar. The permeates (F) and retentates (O) were freeze-dried and analyzed for C_org._, N_tot._, P_tot._ and carbohydrate contents, and used for FTIR analyses.

For enzymatic study, the macroaggregate interstitial water used as a substrate was firstly filtered through 0.7 μm pore size Whatman GF/F filters and then filtered through a 0.22 μm pore size Nucleopore filter and, finally, separated by ultrafiltration as described above. The obtained retentates were subsequently hydrolyzed using α-amylase and β-glucosidase at 26 °C, the mean summer surface temperature in the Gulf of Trieste, for 14 days, and the products freeze-dried and analyzed for C_org._, N_tot._ and total carbohydrate content, and characterized using FTIR analyses. Concentration of both enzyme stock solutions was 1 mg/mL. All experiments were performed in triplicate.

### 4.3. Analyses

C_org._ and N_tot._ in freeze-dried samples were analyzed using a Carlo Erba mod. EA 1108 C, H, N, S analyzer and P_tot._ colorimetrically [44] after sample digestion with K_2_S_2_O_8_. Total proteins, carbohydrates and lipids were assayed colorimetrically using the Coomassie Brilliant Blue method of Setchell *et al.* [45], the phenol-sulphuric acid method of Dubois *et al.* [46] and the Folch [47] method, respectively. Standards comprised solutions of d-glucose (Sigma) for carbohydrates and BSA (Sigma) for proteins in Milli-Q water. All colorimetric analyses were performed in triplicate. FTIR spectra were obtained from homogenized samples using a Perkin-Elmer Spectrum One spectrometer with a diffuse reflectance sampling accessory. The micro-cup of the accessory was filled with the sample diluted by anhydrous KBr to give up to a 5% mixture. Spectra were collected at room temperature with a resolution of 4 cm^−1^ and 4–10 scans were accumulated for each spectrum in a frequency range of 4000–450 cm^−1^.

## 5. Conclusions

Results obtained from the enzymatically hydrolyzed macroaggregate matrix, prevalently of phytoplankton origin, by amylase, glucosidase, protease, proteinase and lipase, reveal fast, almost simultaneous, decomposition of polysaccharides and proteins, but slower decomposition of lipids probably in the form of glycolipids. Hence, the majority of carbohydrate and protein pools are potentially degradable, while the great majority of lipids can be preserved in the water column and transported away and finally deposited on the seabed. A pronounced degradation of the α-glycosidic bond compared to β-linkages was observed, probably due to the presence of α-reserve algal polysaccharides. The rapid hydrolysis of the organophosphorus component suggests fast cycling of macroaggregate phosphorus.

Hydrolysis of the macroaggregate matrix with α-amylase + β-glucosidase resulted in a release of inorganic particles, indicating an important interaction between carbohydrates and minerals. Minerals act as an important aggregation nucleus in mucilage formation and stabilization.

*N*-containing polysaccharides seem to be important constituents of the higher MW colloidal fractions, whereas poly- and oligosaccharides prevailed in the lower MW fractions. Degradation of the polysaccharide component proceeded faster in the higher MW fractions contributing to the preservation of organic nitrogen in the form of less degradable *N*-containing polysaccharides.

Our present knowledge indicates that various macroaggregate fractions and components are subjected to compositionally selective lability with important implications for macroaggregate persistence.

The compositional and degradation studies of marine mucous macroaggregates can contribute to the general knowledge of gels, gelation and aggregation (sol-gel transition) processes, binding capacity and other adsorbing properties, ion-exchange processes and inter- and intra-associations of such substrates, and the outcomes can potentially be used for technological purposes. To our knowledge, there are no reports on technological and pharmaceutical use of marine macroaggregates.
